# A Methodological Framework to Discover Pharmacogenomic Interactions Based on Random Forests

**DOI:** 10.3390/genes12060933

**Published:** 2021-06-18

**Authors:** Salvatore Fasola, Giovanna Cilluffo, Laura Montalbano, Velia Malizia, Giuliana Ferrante, Stefania La Grutta

**Affiliations:** 1 Institute for Biomedical Research and Innovation, National Research Council, 90146 Palermo, Italy; giovanna.cilluffo@irib.cnr.it (G.C.); laura.montalbano@irib.cnr.it (L.M.); velia.malizia@irib.cnr.it (V.M.); stefania.lagrutta@irib.cnr.it (S.L.G.); 2 Department of Health Promotion, Mother and Child Care, Internal Medicine and Medical Specialties, University of Palermo, 90127 Palermo, Italy; giuliana.ferrante@unipa.it

**Keywords:** cancer, cell lines, drug response, genomic alterations, pharmacogenomic interactions, Random Forests

## Abstract

The identification of genomic alterations in tumor tissues, including somatic mutations, deletions, and gene amplifications, produces large amounts of data, which can be correlated with a diversity of therapeutic responses. We aimed to provide a methodological framework to discover pharmacogenomic interactions based on Random Forests. We matched two databases from the Cancer Cell Line Encyclopaedia (CCLE) project, and the Genomics of Drug Sensitivity in Cancer (GDSC) project. For a total of 648 shared cell lines, we considered 48,270 gene alterations from CCLE as input features and the area under the dose-response curve (AUC) for 265 drugs from GDSC as the outcomes. A three-step reduction to 501 alterations was performed, selecting known driver genes and excluding very frequent/infrequent alterations and redundant ones. For each model, we used the concordance correlation coefficient (CCC) for assessing the predictive performance, and permutation importance for assessing the contribution of each alteration. In a reasonable computational time (56 min), we identified 12 compounds whose response was at least fairly sensitive (CCC > 20) to the alteration profiles. Some diversities were found in the sets of influential alterations, providing clues to discover significant drug-gene interactions. The proposed methodological framework can be helpful for mining pharmacogenomic interactions.

## 1. Introduction

Mining pharmacogenomic interactions in cancer research is of crucial importance for identifying the profiles of patients who are most likely to benefit from specific therapies [1]. In this regard, the Cancer Cell Line Encyclopedia (CCLE) [2] and the Genomics of Drug Sensitivity in Cancer (GDSC) [3] projects have screened large panels of cancer cell lines using multiple drug candidates, unveiling several known and novel biomarkers of drug sensitivity [4]. In particular, genomic alterations, including somatic mutations and copy number changes (gene amplifications and deletions), are increasingly being considered as candidate biomarkers of drug sensitivity [5]. However, complex interactions involving combinations of genomic alterations may be associated with drug response [6].

Accurate quantification of drug cytotoxicity is crucial in precision medicine for cancer, and different statistical methods and metrics have been developed based on dose-response curve characteristics [7]. Among them, the area under the dose-response curve (AUC) has been recommended [8].

The conventional definition of “pharmacogenomic interaction” in the relevant literature refers to the situation in which genomic features (*X*, *Z*) are associated with the response to a given drug (*Y_d_*) across a set of screened cell lines (*Y_d_ ~ X + Z*). Although usually disregarded, this is in line with the statistical definition of interaction, i.e., the situation in which the effect of the drug used (*d*) on a cytotoxicity indicator (*Y*) depends on the genomic features of the target cell lines (*Y ~ d × [X + Y]*). A natural way of obtaining clues about the presence of drug-gene interactions is indeed estimating independent models for several compounds (*Y_d_ ~ X + Z*) and seeking differential associations [9].

A broad range of supervised machine learning algorithms is available for predicting drug sensitivity in precision oncology applications [10]. In particular, Elastic Net regression [11] and Random Forests [12] have been recommended due to their good predictive performances [13]. Other approaches adopted in recent studies analyzing data from CCLE and/or GDSC include drug-gene common module identification methods (based on non-negative matrix factorization, partial least squares, and network analysis) [14], mutation pair models (based on linear regression) [15], drug-gene similarity network models [16], and Bayesian regression [17]. Although all the aforementioned approaches have been demonstrated to ensure good predictive performances, several concerns may limit their applicability in pharmacogenomic studies: computational complexity, assumption validity (e.g., linearity), tuning parameter selection, interpretability, ability to handle numerical/categorical variables, and ability disentangle the importance of single features or their combinations.

In this regard, Random Forests are quite flexible, assumption-free, and able to incorporate the effect of predictor combinations (*Y_d_ ~ X × Z*) as a natural consequence of their tree structure [18]. On the other side, Random Forests may be lacking in terms of interpretability, and their computational burden (in terms of both time and required memory) rapidly grows with the number of samples and predictors. Therefore, the estimation of multiple Random Forests may become unfeasible on ordinary computer platforms.

In 2010, Riddick et al. developed a multistep algorithm for predicting in vitro drug response from gene expression data, showing that Random Forests yield superior predictive accuracy to univariate or additive models [19]. This algorithm is able to create drug-specific gene expression signatures and to identify core cell lines involved in the associations [19]. A similar algorithm has not been developed for mutation data and for mining drug-gene interactions in a statistical fashion.

In this study, we aimed to provide a methodological framework for mining pharmacogenomic interactions based on Random Forests. The proposed methodology is thought to be carried out with ordinary computational resources and using R version 4.0.2 (R Foundation for Statistical Computing, Vienna, Austria) as the reference software. A comprehensive source code is provided in the Supplementary Material, including indications about all the required libraries and some data not shown.

## 2. Materials and Methods

### 2.1. Alteration and Response Datasets

In the current study, we considered two publicly available datasets from the CCLE and the GDSC projects. Thereafter, they will be referred to as the “Alteration” dataset and the “Response” dataset. Both datasets were accessed on 1 February 2021.

The CCLE Alteration dataset (CCLE_MUT_CNA_AMP_DEL_binary_Revealer.gct) was downloaded from: https://portals.broadinstitute.org/ccle/data (database file date: 29 February 2016). This dataset contains 48,270 rows corresponding to the same amount of possible gene alterations, labelled by the gene name followed by “_MUT” (somatic mutation), “_DEL” (deletion) or “_AMP” (amplification). The 1030 columns correspond to as many tumor tissues (cell lines), labelled by the sample name followed by “_” and the name of the organ involved. The generic entry of the alteration database is a binary indicator assuming value 1 if a given alteration is present in a given cell line and 0 otherwise. There are no missing values in the Alteration dataset.

The GDSC Response dataset (TableS4B.xlxs) was downloaded from: www.cancerrxgene.org/gdsc1000/GDSC1000_WebResources/Home.html (database file date: 7 July 2016). The transpose dataset contains 265 rows corresponding to as many pharmacological compounds, labelled by the drug identifiers. The 990 columns correspond to the same amount of tumor tissues (cell lines), labelled by the sample name. The generic entry of the response database is the AUC for a given cell line and a given drug. The AUC is reported as a fraction of the total area between the highest and lowest screening concentration, ranging from 0 (highest cytotoxicity) to 1 (lowest cytotoxicity). Missing data are present. The two datasets were matched by column, for a total of 648 shared cell lines.

### 2.2. Random Forests

Random Forests [18] are very popular in the field of Machine Learning. A Random Forest is an ensemble of decision trees trained on different bootstrap samples drawn from the same training set. The trained forest is used to predict the response variable for new input data, by averaging the predictions obtained from each individual tree. This allows working around the problem of overfitting that may characterize a single, deep decision tree, especially when the number of predictors is large. Moreover, to reduce the correlation among the trees, a random subset of candidate predictors is selected at random before performing any step of data split. Indeed, by reducing the redundancy among the trees, predictive performances are further improved. Random Forests can satisfactorily deal with both numerical and categorical outcome/predictors. As detailed in Section 2.4 and in Section 2.5, Random Forests are able to efficiently provide reliable indicators of predictive performance and variable importance.

Random Forests have two tuning parameters. The first one is the number of trees in each forest, say *B*. For this parameter, a value of 500 (*R* default) can be sufficiently large to attain model stability. In this study, a stability check was performed ex-post by calculating the mean of the last ten squared differences in the prediction error through the forest growth process. The second tuning parameter is the number of candidate predictors to select at random before each data split, say *m*. Its optimal selection would require cross-validation, but this would become computationally prohibitive in our context (because we have to fit 265 models). Many researchers have generally used “one-third of the predictors” as the default choice; this is also the default in the *R* package *randomForest* [20], and fair predictive performances have been obtained using this value [21].

In the present study, Random Forests allowed us to satisfactorily cope with the following aspects: (1) the number of predictors is large, possibly leading to overfitting concerns; (2) predictors are binary; (3) outcomes are continuous; (4) we are interested in assessing the extent to which each drug is sensitive to gene alteration profiles; (5) we want to avoid cross-validation for saving computational time; (6) we are interested in assessing variable importance, possibly through a *p*-value. 

### 2.3. Data Reduction

Here we propose a three-step data reduction aiming to save computational costs. The first step was performed by selecting alterations involving genes included in a list of 568 genes previously identified as cancer drivers [22]. The full list can be downloaded at the following URL: https://www.intogen.org/download (accessed on 1 February 2021).

The second step stems from considering that alterations that are always or never observed in the database (0 variance) will never be included in the Random Forests. Similarly, very frequent/infrequent alterations (low variance) will be less likely to be included in the forests. Given the alteration proportion, say *p* (relative frequency), the alteration variance was derived as *p* × *(1 − p)* (the variance of a binary variable). A “low” variance was set by specifying a small proportion, i.e., 0.05, and calculating the corresponding variance as 0.05 × 0.95 = 0.0475. All the alterations with a variance below the threshold were excluded from the analyses.

The last step stems from considering that Random Forests tend to level the importance of highly correlated (redundant) alterations [23]. In this sense, we propose to apply a hierarchical clustering of predictors [24] to identify groups of correlated alterations and to reduce the redundancy of information by keeping a single representative for each group. In particular, we used a complete-linkage clustering and one minus the squared Pearson correlation matrix of the alterations as the distance matrix. A “small” distance was set by specifying a high correlation, i.e., 0.95, and calculating the corresponding distance as 1 − 0.95^2^ = 0.0975. After cutting the dendrogram at the aforementioned small distance, we stored the original cluster composition and then retained the first alteration in each cluster.

### 2.4. Predictive Performance

Random Forests provide a convenient way for assessing the “out-of-bag” (OOB) predictive performance of the model without the need to perform cross-validation. First, each response (the AUCs, say $Y_{i}$) is predicted by using the subset of trees (say $B_{i}$) trained without that observation:

(1) $${\hat{Y}}_{i}^{OOB}=\frac{1}{\left|B_{i}\right|}\sum_{b\in B_{i}}{\hat{Y}}_{i}^{b}$$

With *B* sufficiently large, it can be shown that the aforementioned OOB predictions are virtually equivalent to leave-one-out cross-validation predictions [25]. The aforementioned vector of OOB predictions could therefore be used to calculate an OOB mean squared error. However, for the sake of comparability between models, we propose to use another measure of agreement, i.e., the concordance correlation coefficient (CCC) [26] between observed AUCs and OOB predictions. The CCC can be calculated as:

(2) $$CCC(Y,{\hat{Y}}^{OOB})=\frac{2\mathrm{cov}(Y,{\hat{Y}}^{OOB})}{\mathrm{var}(Y)+\mathrm{var}({\hat{Y}}^{OOB})+{\left(\bar{Y}-{\bar{Y}}^{OOB}\right)}^{2}}$$

The CCC ranges between −1 and 1, and it is more conservative than a Pearson correlation (it is 1 only if the two vectors are identical). In this study, the CCCs were multiplied by 100. The following benchmarks were used to qualify the concordance: ≤0, “none”; 1 to 20, “poor”; 21 to 40, “fair”; 41 to 60, “moderate”; 61 to 80, “substantial”; 81 to 100, “excellent” [27]. A 95% confidence interval (CI) was obtained, and a lower limit of lower CCC > 20 was used to qualify an at least fair concordance.

### 2.5. Variable Importance

The importance of each predictor (alteration, say $X_{j}$) in each forest was quantified by the permutation importance [28], i.e., the mean change of the prediction error in the OOB samples of each tree (say $OOB_{b}$) after random permutation of that predictor (say ${\tilde{X}}_{j}$):

(3) $$imp(X_{j})=\frac{1}{B}\sum_{b=1}^{B}\left[\frac{\sum_{i\in OOB_{b}}{\left[Y_{i}-{\hat{Y}}_{i}({\tilde{X}}_{j})\right]}^{2}}{\left|OOB_{b}\right|}-\frac{\sum_{i\in OOB_{b}}{\left[Y_{i}-{\hat{Y}}_{i}(X_{j})\right]}^{2}}{\left|OOB_{b}\right|}\right]=\frac{1}{B}\sum_{b=1}^{B}\left(imp_{j}^{b}\right)$$

The importance indicators were then normalized through their estimated standard deviations to obtain an approximate z-score [29] as:

(4) $$z_{j}=\frac{imp(X_{j})}{\sqrt{\frac{\sum_{b=1}^{B}{\left[imp_{j}^{b}-imp(X_{j})\right]}^{2}}{B-1}}}$$

Finally, a *p*-value for testing the null hypothesis of no importance was derived, for each alteration, as the areas under the normal curve to the right of $z_{j}$. Within each model, a Bonferroni correction was applied to the *p*-value vector, and a given alteration was deemed as significantly influential if *p* < 0.005, i.e., a more conservative criterion (than *p* < 0.05) that has been endorsed for claims of new discoveries [30].

### 2.6. Missing Values

In GDSC, not all the cell lines were screened for each pharmacological compound. Therefore, before estimating each model, cell lines with missing AUCs were not included in the Random Forest. This may alter the distribution of alteration variances, as well as their pairwise correlations. In particular, they may violate the thresholds set. Therefore, for each model, we checked the CCC between the original and altered variance/squared correlation distribution, and the frequency of violated thresholds (low variances and high correlations), by plotting them against the sample size.

### 2.7. Reporting Results

After presenting summary statistics for the two datasets, we reported elapsed times for the 265 Random Forests by sample size and the stability indicator distribution. Then, we reported the frequency distribution of the prediction CCC across the models by class. We also investigated the possible effects of sample size and average compound AUC on the CCC. Therefore, we produced two reports. Report 1 lists the compounds associated with an at least fair CCC (lower CCC > 20), in decreasing order of CCC, and for each of them: the CCC and its 95% CI, mean AUC, sample size, number and name of significantly influential alterations in decreasing order of importance. Report 2 lists the alterations that influence the compounds in Report 1 in decreasing order of significance frequency and for each of them: ID and size of the corresponding alteration cluster, names of influenced compounds in decreasing order of CCC.

### 2.8. Discovering Drug-Gene Interactions

The two reports were used for obtaining clues about the presence of drug-gene interactions. In particular, we carried out further investigations for pairs of compounds with similar average AUCs but different, very influential alterations. In this case, after logit normalization of the AUC, we performed a graphical investigation by plotting the overall logit (AUC) distribution for the two compounds, and the logit(AUC) distributions conditional to combinations of the two alterations. We also performed a formal test for interaction through a two-way ANOVA with logit (AUC) as the response, the compound as Factor 1, and the alteration combination as Factor 2.

A comprehensive methodological workflow about the present work is provided in the Supplementary Material.

## 3. Results

**Figure 1:**
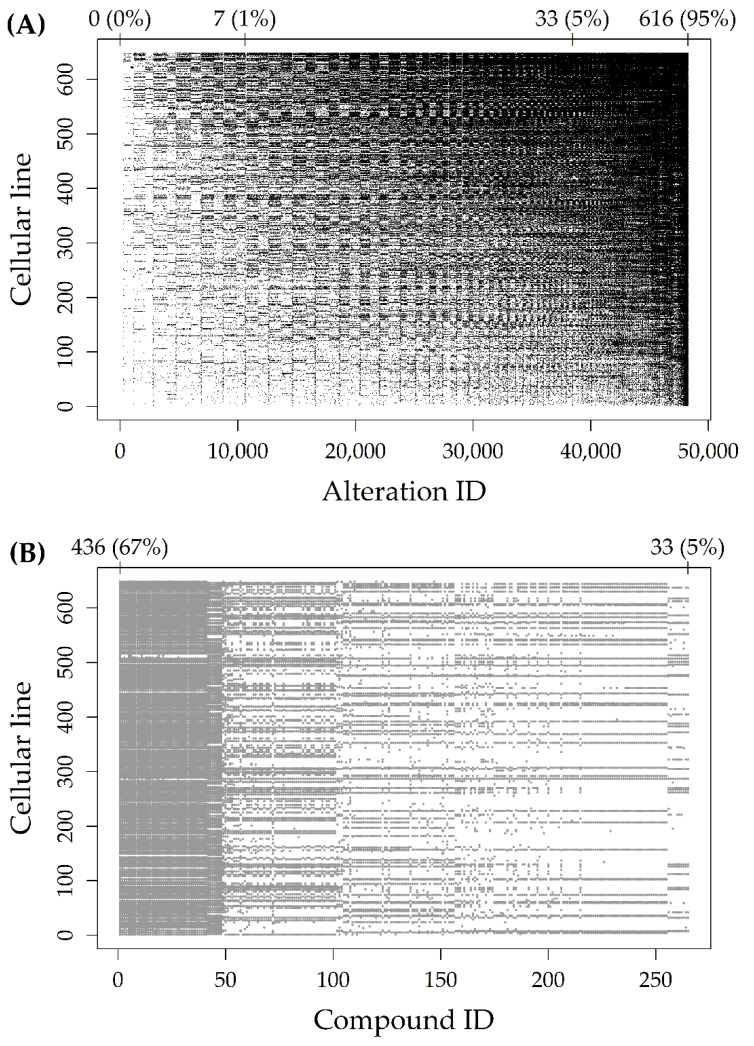
(**A**) Alteration dataset with the 48,270 rows (alteration types, reported on the *x*-axis) and the 648 columns (cell lines, reported on the *y*-axis) in increasing order of alteration frequency. Black dots indicate altered cells. Frequency (percentages) above the plot indicate row positions at which those alteration frequencies are reached for the first time; (**B**) Response dataset with the 265 rows (compounds, reported on the *x*-axis) in increasing order of sample size, and the 648 columns (cell lines, reported on the *y*-axis) in increasing order of alteration frequency. Grey dots indicate missing AUCs. The two frequencies (percentages) above the plot indicate the largest and smallest number of missing AUCs, respectively.

Figure 1A represents the Alteration dataset, with black dots indicating altered cells. The 48,270 rows (alteration types, reported on the *x*-axis) and the 648 columns (cell lines, reported on the *y*-axis) were reported in increasing order of alteration frequency. As indicated by the numbers above the plot, there were about 10,000 rows with less than 1% (7/648) of altered cell lines. Moreover, there were about 40,000 rows with less than 5% (33/648) of altered cell lines. Figure 1B represents the Response dataset, with grey dots indicating missing AUCs. The 265 rows (compounds, reported on the *x*-axis) were reported in increasing order of sample size, while the 648 columns (cell lines, reported on the *y*-axis) were reported in increasing order of alteration frequency (as in Figure 1A). As indicated by the numbers above the plot, the proportion of missing AUCs ranged from 67% (436/648) to 5% (33/648), for an average sample size of 523 cell lines.

A total of 2567 alteration types involved one of the 568 driver genes. Among them, 1990 had a variance below the threshold and were excluded. Clustering the 577 remaining predictors identified 501 alteration groups (447 of size 1), and the 501 corresponding representatives were included in the final analyses. The most frequent tissues of origin of the 648 cell lines were the lung and the hematopoietic and lymphoid tissue (Table 1).

**Figure 2:**
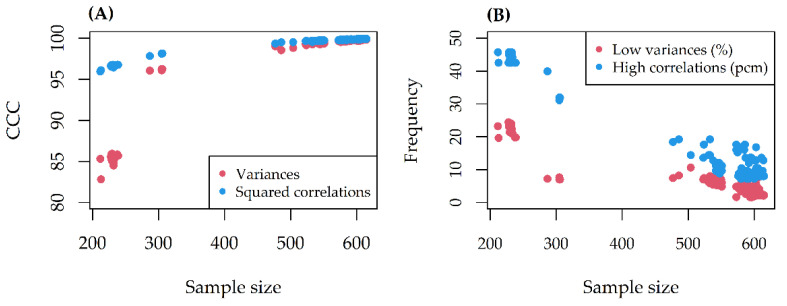
(**A**) Concordance correlation coefficient (CCC) between alteration variances/squared correlations before and after missing data removal, as a function of sample size; (**B**) Frequency of variances/correlations violating the thresholds set, as a function of sample size.

The CCC between alteration variances/squared correlations before and after missing data removal was always excellent (>80) (Figure 2A). At most, 25% of alteration variances and 45 pcm (percent mille, or per hundred thousand) of pairwise correlations violated the thresholds set (Figure 2B). The missing effect decreased as the sample size increased.

**Figure 3:**
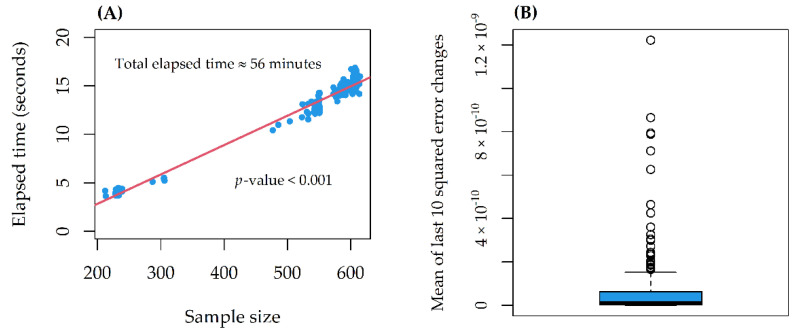
(**A**) Computational times elapsed as a function of sample size. The *p*-value is from linear regression (red line); (**B**) Distribution of the stability indicator through the 265 models.

**Figure 4:**
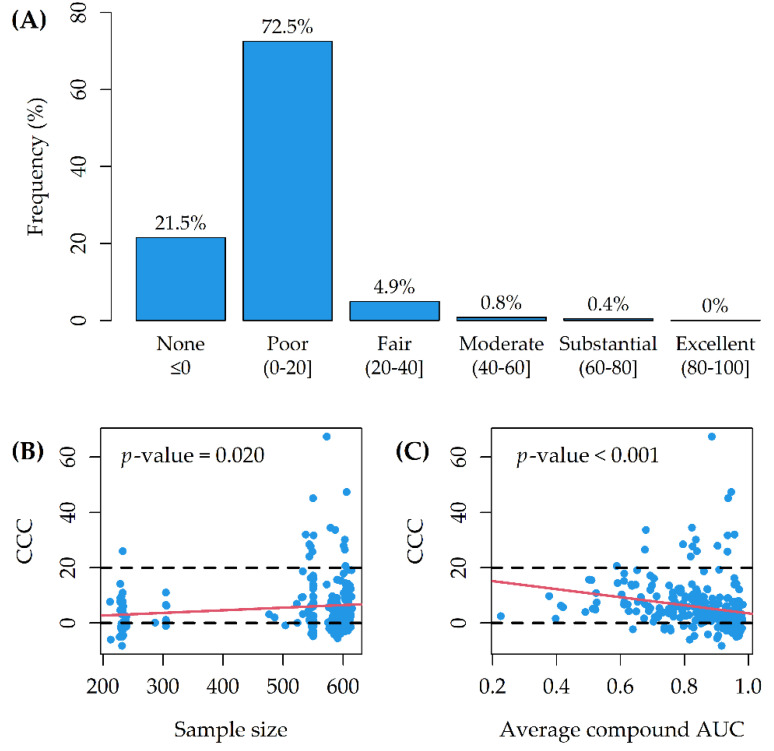
(**A**) Concordance correlation coefficient (CCC) distribution through the 265 Random Forests; (**B**) CCC as a function of sample size; (**C**) CCC as a function of average compound AUC. The *p*-values are from linear regressions (red lines). Dashed lines correspond to the thresholds of no concordance (CCC = 0) and fair concordance (CCC = 20).

On a 10th generation Core i7 with 4 cores (up to 3.9 GHz) and 16 GB SDRAM DDR4, the computational time increased linearly with the sample size, for a total of 56 min (Figure 3A). The stability indicator was 0 (stability reached) in all models (Figure 3B). For 16/265 (6%) of the Random Forests, the CCC between observed and predicted AUCs was larger than 20 (lower CCC > 20 for 12 of them) (Figure 4A). On average, model CCCs were tendentially larger as the sample size increased (Figure 4B), while they were tendentially smaller as the average compound AUC increased (Figure 4C).

Table 2 shows Report 1 up to the two most influential alterations for each compound; Supplementary Table S1 shows the whole of Report 1. Dabrafenib was associated with the largest CCC (67.4, 95% CI: 63.1, 71.3), while (5Z)-7-Oxozeaenol was associated with the smallest CCC (26.5, 95% CI: 21.6, 31.2) (Table 2). In the whole of Report 1, Trametinib was associated with the largest number (16) of significantly influential (*p* < 0.005) alterations (Supplementary Table S1). BRAF mutations were strong predictors for Dabrafenib, PLX4720, and SB590885. BRAF and KRAS mutations were strong predictors for RDEA119, Trametinib, AZD6244, and PD-0325901. BRAF and NRAS mutations were strong predictors for (5Z)-7-Oxozeaenol. TP53 mutation and MAP2K4 deletion were strong predictors for Nutlin-3a. IKZF3 and ERBB2 amplification were strong predictors for Afatinib (rescreen).

Table 3 shows Report 2 up to alterations significantly influential (*p* < 0.005) for >1 compounds; Supplementary Table S2 shows the whole of Report 2. BRAF.V600E_MUT and BRAF_MUT alterations were significantly influential for the largest number of compounds in Report 1 (11 and 10, respectively).

**Figure 5:**
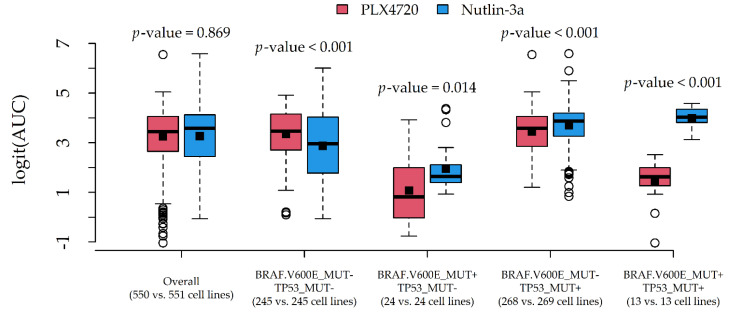
Graphical inspection of a drug-gene interaction involving the two compounds PLX4720 and Nutlin-3a, and the two alterations BRAF.V600E_MUT and TP53_MUT. Boxplots represent the median (central line), the mean (square), 25th–75th percentiles (box), and min-max non-outlier values (whiskers); *p*-values are from the *t*-test.

**Figure 6:**
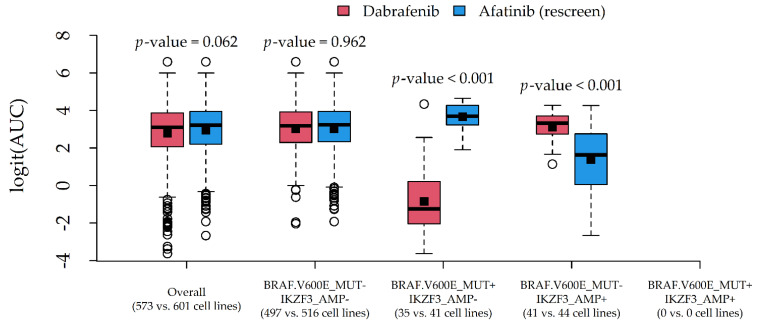
Graphical inspection of a drug-gene interaction involving the two compounds Dabrafenib and Afatinib (rescreen), and the two alterations BRAF.V600E_MUT and IKZF3_AMP. Boxplots represent the median (central line), the mean (square), 25th–75th percentiles (box), and min-max non-outlier values (whiskers); *p*-values are from the *t*-test.

Two couples of potential drug-gene interactions deserved further investigation. The first interaction (Figure 5) involved PLX4720 and Nutlin-3a, due to a very similar average AUC (0.937 and 0.936) and different most influential alterations (BRAF.V600E_MUT and TP53_MUT). The second interaction (Figure 6) involved Dabrafenib and Afatinib (rescreen), due to a similar average AUC (0.886 and 0.904) and different most influential alterations (BRAF.V600E_MUT and IKZF3_AMP).

In both cases, the average response difference between the two compounds was related to the particular combination of alteration status, which was suggestive of statistical drug-gene interaction. A formal test via two-way ANOVA indeed confirmed the presence of statistically significant interaction effects (*p*-values < 0.001).

## 4. Discussion

We provided a statistical methodological framework for mining and graphically exploring drug-gene interactions based on Random Forests. After three steps of data reduction, several models were fitted using the AUC as the drug sensitivity indicator and copy number and mutation data as the predictors. Then, we used known statistical indicators of model predictive performance and variable importance and produced tabular and graphical reports of the results. Using an ordinary computer platform and the R software, we identified 12 compounds associated with an at least fair concordance between observed AUCs and OOB predictions, in a reasonable computational time (56 min). Moreover, some diversities were found in the sets of influential alterations, providing clues to discover significant drug-gene interactions.

The OOB predictive performance of the models was poor for 72.5% of the tested drugs (Figure 4A). Indeed, the information content of genomic data (somatic mutations and copy number changes) is known to be lower than, for example, gene expression data in the pan-cancer setting [4,31]. Copy number changes may be associated with each other, and mutations may characterize only a few genes [13]. On the other side, genomic data can more easily translate into clinical biomarkers, as a consequence of the increased molecular stability of DNA compared to RNA [13]. Moreover, genomic alterations are more likely to represent functional (causal) drivers of drug sensitivity [13].

Model CCCs were tendentially larger as the number of cell lines tested with each drug increased (Figure 4B); indeed, smaller sample sizes may have straightforwardly influenced the predictive performance for several models. Moreover, the CCCs were tendentially smaller as the average compound AUC increased (Figure 4C). This is consistent with the finding of an increasing estimation uncertainty of the AUC for experiments with a partial response (AUC between 0.4 and 0.9) [32]. In general, while using the AUC for assessing drug sensitivity has been shown to increase the predictive performances in pharmacogenomic models [13], using a single summary statistic may not be optimal [33]. In this regard, the use of multivariate analysis of variance (MANOVA) has been endorsed for the joint modelling of multiple drug sensitivity indicators (associated with individual genomic features), such as the traditional metric of IC_50_ (the concentration at which the compound reaches 50% reduction in cell viability) and the slope of the dose-response curve [3].

The proposed methodological approach may appear simplistic or potentially affected by workarounds aiming to save computational time (especially data reduction). Nevertheless, we were able to detect well-known associations (Supplementary Table S1) such as Dabrafenib-BRAF (sensitivity, Figure 6) [34], Nutlin-3a-TP53 (resistance, Figure 5) [35], Afatinib-ERBB2 and Afatinib-EGFR (sensitivity, data not shown) [36,37], and other associations reported by the Drug–Gene Interaction Database (DGIdb 4.0) [38]. Potentially novel associations were also found, such as Afatinib-IKZF3 (sensitivity, Figure 6) and Nutlin-3a-MAP2K4 (resistance, data not shown), which need, however, to be validated in in vivo models. Although we conservatively limited our reports to drugs with an at least fair CCC (>20), the provided R code (Supplementary Materials) allows users to reduce this threshold (e.g., >10) to include less predictable compounds in the reports, and to obtain more (but less precise) clues about potential drug-gene interactions. 

In this regard, we also provided clues to producing graphical reports (Figure 5 and Figure 6) for exploring drug-gene interactions. This was accomplished by considering pairs of compounds with similar AUCs but different associated alterations, and plotting their (logit) AUC distributions against combinations of influential alterations. Although AUC comparisons among drugs may be hazardous, such investigation is more in line with the statistical definition of interaction, i.e., the situation in which drug effects on a cytotoxicity indicator depend on the genomic features of the target cell lines. In this novel perspective, a formal test for statistical interactions was performed via two-way ANOVA with (logit) AUC as the response, compound as Factor 1, and alteration combination as Factor 2.

Among available Machine Learning algorithms, we used Random Forests. Random Forests have already been applied in previous genomic studies [10], showing high predictive accuracy at the expense of model interpretability [13]. Although Elastic Net regression has been recommended as a valid (or even better) alternative [13], its use was not fully indicated in the current framework. In particular, Elastic Net regression has two tuning parameters for the Ridge/Lasso contributions (no defaults have been proposed), requires a standardization of the (quantitative) features, and does not provide *p*-values for statistical testing of feature importance. Moreover, Elastic Net regression does not automatically account for the effect of feature combinations, does not provide natural alternatives to cross-validation, and requires a test set for the assessment of predictive performance. Logic Regression [4] is another elegant solution to evaluate predictor combinations but, differently from Random Forests, it requires tuning model complexity (number of combinations/alterations involved) and would not be extensible to continuous predictors such as gene expression. Discovering markers using multiple statistical tests has also been recommended [39]; however, as for MANOVA, it involves testing one genomic feature at a time, overlooking feature combinations.

Estimating multiple Random Forests requires time and memory resources, especially with many predictors. In particular, trying to estimate a single Random Forest with the original 48,270 alterations rapidly saturated the system in an ordinary computer platform (10th generation Core i7, 4 cores up to 3.9 GHz, 16 GB SDRAM DDR4). For this reason, we proposed three steps of data reduction: limiting to driver genes, excluding frequent/infrequent alterations, and excluding redundant alterations. It is worth noting that, differently from usual feature selection algorithms, the proposed data reduction is performed before the predictors could see the responses, so that overfitting is prevented.

Although limiting to driver genes may lead to overlooking potentially unexplored associations, it has been observed that mutations on driver genes can be responsible for both the genesis and the course of malignancies, including drug sensitivity [40,41]. 

Similarly, excluding infrequent alterations of driver genes may lead to disregarding potentially important biomarkers of response to therapies [42]. It should be noted that, in our application, the term “infrequent” refers to the frequency observed in the specific dataset. Conversely, the term “rare” would be more appropriate for referring to general cell line populations [43] (the two terms are associated but not identical). Although we set a proportion equal to 0.05 (or 0.95) to denote an infrequent (or frequent) alteration, this parameter can be decreased or even increased according to the available hardware. As a raw check of statistical power, given the average sample size (523), the *t*-test for alterations with a relative frequency of 0.05 (or 0.95), a significant level of 0.05, and a moderate effect size [44] has a power of about 0.70. This may be acceptable, especially if we assume that statistical testing through Random Forest ensures higher power [28]. To get a power of at least 0.80, the small frequency should be set at 0.065 (see the R code provided in the supplement for calculations).

In the last step of data reduction, we applied a hierarchical clustering of predictors to reduce the redundancy of information by keeping a single representative for each group. Because the original cluster composition is stored, excluded (redundant) alterations are not completely discarded with this reduction step. They will simply be considered as being as important as their representatives. Although we set a correlation (notice that Pearson correlation equals Cramer’s V for binary variables) equal to 0.95 (or −0.95) to qualify redundant (or specular) alterations, this parameter can also be modified according to the available hardware. In particular, decreasing the threshold (e.g., to 0.8) would produce fewer alteration clusters and, consequently, fewer representatives.

### 4.1. Strengths

The main strength of the proposed methodological framework is its relative simplicity, computational efficiency, and flexibility. Indeed, the methodology can be extended to consider other predictor categories, such as gene expression and DNA methylation, and/or different drug sensitivity indicators. Moreover, we gathered data from two publicly available large-scale pharmacogenomics resources, the CCLE and the GDSC. For these projects, substantial agreement has been observed in the provided measurements of drug sensitivity and genomic predictors [45], and powerful connectivity tools have been developed [46]. Finally, we applied reliable statistical methodologies for assessing feature importance (permutation importance and the associated *p*-value) and model predictive performance (CCC). In particular, differently from previous studies [4,12,16], we used the more conservative CCC rather than the Pearson correlation coefficient for assessing the agreement between observed and predicted drug responses. These tools simplified the creation and interpretation of tabular and graphical reports.

### 4.2. Limitations

Several limitations should also be acknowledged. First, as previously mentioned, the proposed methodological approach is affected by workarounds and somewhat arbitrary choices aimed at saving computational time, especially thresholds for data reduction and the use of default tuning parameters for the Random Forests. Another concern is that several missing data were removed. In particular, because the sets of cell lines with unavailable AUCs were different for different drugs, this may have affected the comparability among different models. In this regard, we provided suggestions to perform graphical checks: a check of model stability (Figure 3B) and a check of missing effects (Figure 2). 

Finally, our analysis was carried out by including all cancer types available in the database (pan-cancer setting). Although this ensures larger sample sizes, between-tissue heterogeneity in both drug response and tumor molecular characteristics may introduce biases into pan-cancer analysis [4]. In this case, possible workarounds may include performing tissue-specific analyses (at the expense of the sample size) or considering Random Forest extensions that are able to incorporate the aforementioned heterogeneity [47].

## 5. Conclusions

In conclusion, this article presented a reliable, flexible, and efficient framework of statistical methodology for mining and graphically exploring drug-gene interactions based on Random Forests. In a reasonable computational time, the proposed methodology allowed us to identify well-known drug-gene associations and provided clues to discover novel pharmacogenomic interactions. An open R code was made available for implementation in ordinary computer platforms.

## Figures and Tables

**Table 1:** Tissues of origin of the 648 cell lines, ordered by frequency.

Tissue	No. (%)
Lung	134 (20.7%)
Hematopoietic and lymphoid tissue	111 (17.1%)
Breast	46 (7.1%)
Large intestine	43 (6.6%)
Central nervous system	39 (6%)
Skin	36 (5.6%)
Ovary	31 (4.8%)
Pancreas	28 (4.3%)
Esophagus	24 (3.7%)
Stomach	22 (3.4%)
Liver	17 (2.6%)
Urinary tract	17 (2.6%)
Upper aero digestive tract	16 (2.5%)
Soft tissue	15 (2.3%)
Kidney	14 (2.2%)
Autonomic ganglia	12 (1.9%)
Bone	11 (1.7%)
Endometrium	10 (1.5%)
Thyroid	9 (1.4%)
Pleura	6 (0.9%)
Prostate	5 (0.8%)
Biliary tract	1 (0.2%)
Small intestine	1 (0.2%)

**Table 2:** Report 1 up to the two most influential alterations for each compound (in decreasing order of CCC).

Compound	CCC (95% CI)	Mean AUC	Sample Size	Number of Influential Alterations	Alteration 1	Alteration 2
Dabrafenib	67.4 (63.1, 71.3)	0.886	573	4	BRAF.V600E_MUT	BRAF_MUT
PLX4720 (rescreen)	47.3 (42.3, 52.1)	0.946	606	5	BRAF.V600E_MUT	BRAF_MUT
PLX4720	45.1 (39.7, 50.2)	0.937	550	8	BRAF.V600E_MUT	BRAF_MUT
RDEA119 (rescreen)	34.5 (29.0, 39.7)	0.824	579	9	BRAF.V600E_MUT	KRAS_MUT
Trametinib	33.6 (27.9, 39.0)	0.680	587	16	BRAF.V600E_MUT	KRAS_MUT
SB590885	32.0 (26.2, 37.5)	0.957	538	3	BRAF.V600E_MUT	BRAF_MUT
Nutlin-3a	31.7 (25.7, 37.4)	0.936	551	10	TP53_MUT	MAP2K4_DEL
AZD6244	30.1 (24.4, 35.5)	0.836	603	6	BRAF.V600E_MUT	KRAS_MUT
RDEA119	28.4 (22.6, 34.0)	0.796	544	7	BRAF.V600E_MUT	KRAS_MUT
Afatinib (rescreen)	27.9 (22.0, 33.6)	0.904	601	6	IKZF3_AMP	ERBB2_AMP
PD-0325901	27.6 (21.7, 33.3)	0.826	546	8	BRAF.V600E_MUT	KRAS_MUT
(5Z)-7-Oxozeaenol	26.5 (21.6, 31.2)	0.677	603	9	BRAF.V600E_MUT	NRAS_MUT

CCC—concordance correlation coefficient between observed AUCs and out-of-bag predictions; CI—confidence interval; AUC—area under the dose-response curve. Alterations are in decreasing order of permutation importance. Clues about interactions are in green and red.

**Table 3:** Report 2 up to alterations significantly influential (*p* < 0.005) for >1 compounds in Report 1 (in decreasing order of CCC).

Alteration	Significance Frequency	Cluster ID	Cluster Size	Compound 1	Compound 2
BRAF.V600E_MUT	11	184	1	Dabrafenib	PLX4720 (rescreen)
BRAF_MUT	10	385	1	Dabrafenib	PLX4720 (rescreen)
NRAS_MUT	6	185	1	RDEA119 (rescreen)	Trametinib
KRAS.G12_13_MUT	5	431	1	RDEA119 (rescreen)	Trametinib
KRAS_MUT	5	464	1	RDEA119 (rescreen)	Trametinib
CREBBP_MUT	4	384	1	Dabrafenib	Trametinib
FHL5_DEL	4	116	1	PLX4720 (rescreen)	PLX4720
BCL9_AMP	3	358	1	RDEA119 (rescreen)	Trametinib
ARHGAP40_AMP	2	303	1	Trametinib	PD-0325901
CCDC66_DEL	2	383	1	Dabrafenib	Nutlin-3a
MAP2K4_DEL	2	437	1	RDEA119 (rescreen)	Nutlin-3a
RAF1_DEL	2	225	1	Trametinib	Afatinib (rescreen)
TP53_MUT	2	501	1	Nutlin-3a	(5Z)-7-Oxozeaenol

## Data Availability

Publicly available datasets were analyzed in this study. The CCLE data can be found here: https://portals.broadinstitute.org/ccle/data, accessed on 1 February 2021. The GDSC data can be found here: www.cancerrxgene.org/gdsc1000/GDSC1000_WebResources/Home.html, accessed on 1 February 2021.

## Supplementary Materials

The following are available online at https://www.mdpi.com/article/10.3390/genes12060933/s1. Table S1: Report 1, including all the influential alterations for each compound (in decreasing order of CCC): CCC—concordance correlation coefficient between observed AUCs and out-of-bag predictions; CI—confidence interval; AUC—area under the dose-response curve. Alterations are in decreasing order of permutation importance. Clues about interactions are in green and red. Table S2: Report 2, including all the alterations significantly influential (*p* < 0.005) for the compounds in Report 1 (in decreasing order of CCC). Others: zip archive with comprehensive R source code; methodological workflow.
