# Supplementary figures and images for: A Methodological Framework to Discover Pharmacogenomic Interactions Based on Random Forests
Source: Genes (Basel). 2021 Jun 18;12(6):933. doi: 10.3390/genes12060933 (PMC8235396; doi:10.3390/genes12060933)

## Data sources and data reduction

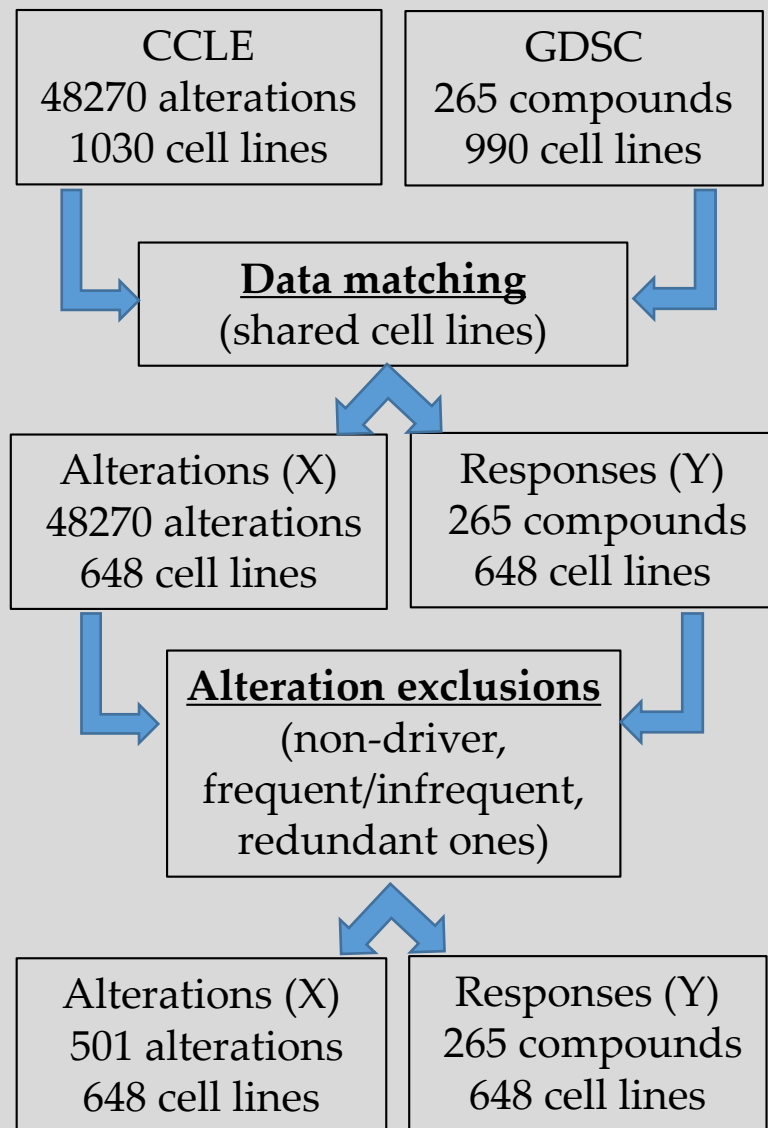

## Data analysis (for each compound)

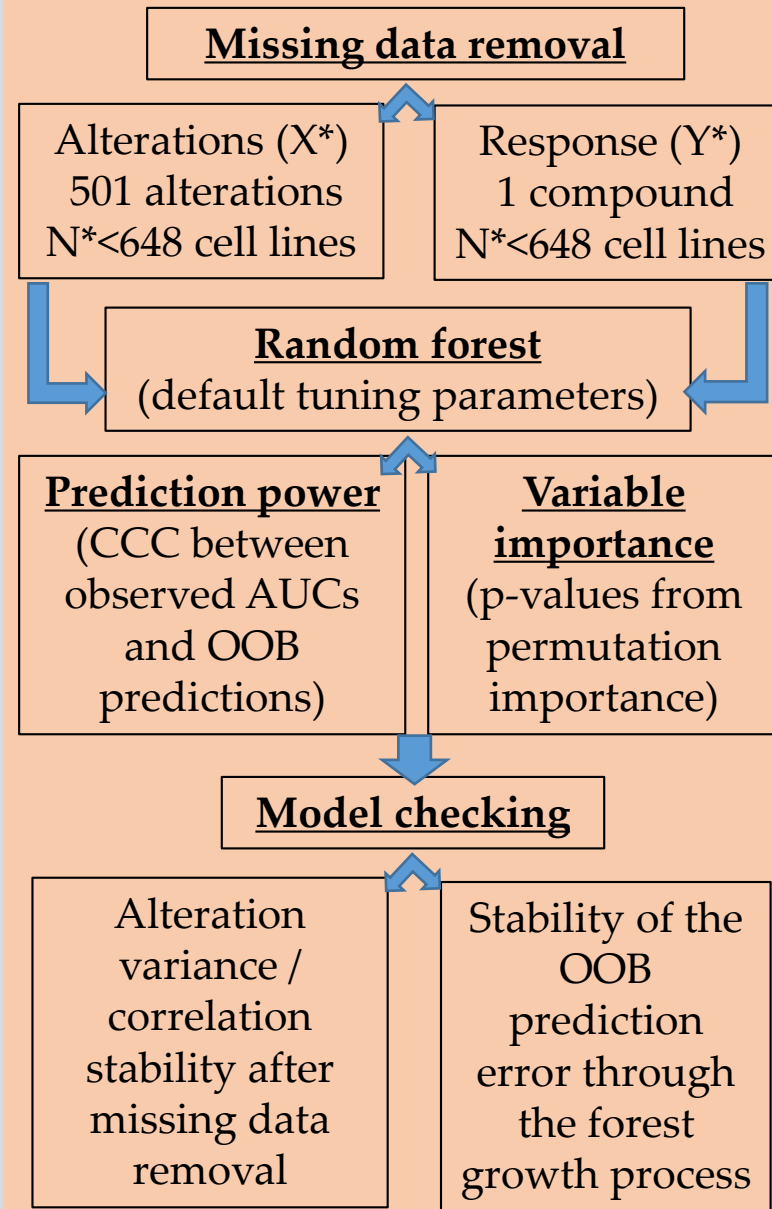

## Reporting results

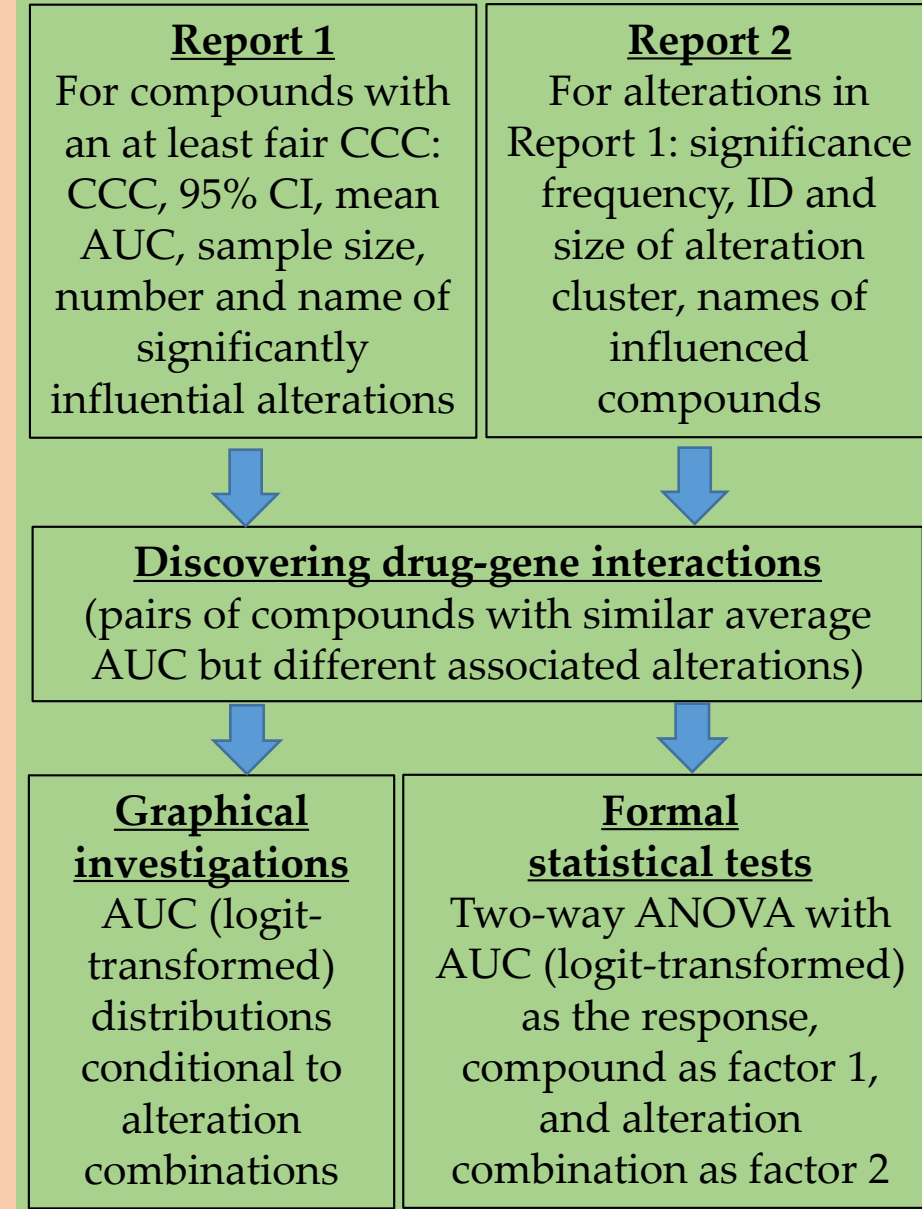

Supplement: Supplementary file 1 [file genes-12-00933-s001.zip › Methodological workflow.pdf]
